# Cardiotoxicity in Elderly Breast Cancer Patients

**DOI:** 10.3390/cancers17132198

**Published:** 2025-06-30

**Authors:** Kalliopi Keramida, Anastasia Constantinidou, Dorothea Tsekoura, Effrosyni Kampouroglou, Chrissovalantis Aidarinis, Emmanouil Saloustros, Georgia Karanasiou, Gaia Giulia Angela Sacco, Erika Matos, Andri Papakonstantinou, Manolis Tsiknakis, Cameron Brown, Athos Antoniades, Carlo Cipolla, Daniela Cardinale, Dimitrios Fotiadis, Gerasimos Filippatos, Investigators CARDIOCARE Consortium

**Affiliations:** 1 2nd Department of Cardiology, Medical School, National and Kapodistrian University of Athens, Athens University Hospital Attikon, 11527 Athens, Greece; 2 Cardiology Department, General Anti-Cancer Oncological Hospital, Agios Savvas, 11522 Athens, Greece; 3 Department of Medical Oncology, Bank of Cyprus Oncology Centre, 32 Acropoleos Avenue, Nicosia 2006, Cyprus; 4 School of Medicine, University of Cyprus, Panepistimiou 1, Nicosia 2408, Cyprus; 5 2nd Department of Surgery, Aretaieio University Hospital, National and Kapodistrian University of Athens, 76 Vas. Sofias Av., 11528 Athens, Greece; 6 Department of Oncology, University Hospital of Larissa, 41110 Larissa, Greece; 7 Unit of Medical Technology and Intelligent Information Systems, University of Ioannina, 45110 Ioannina, Greece; 8 European Institute of Oncology, Istituto di Ricovero e Cura a Carattere Scientific (IRCCS), 20141 Milan, Italy; 9 Department of Medical Oncology, Institute of Oncology Ljubljana, 1000 Ljubljana, Slovenia; ematos@onko-i.si; 10 Department of Breast, Endocrine Tumors and Sarcoma, Karolinska Comprehensive Cancer Center, Karolinska University Hospital, 17164 Solna, Sweden; andri.papakonstantinou@ki.se; 11 Department of Oncology-Pathology, Karolinska Institutet, 17176 Stockholm, Sweden; 12 Department of Electrical and Computer Engineering, Hellenic Mediterranean University, 71410 Heraklion, Greece; 13 Stremble Ventures Ltd., 59 Christaki Kranou, Limassol 4042, Cyprus; cameron.brown@stremble.com (C.B.); athos.antoniades@stremble.com (A.A.); 14 Cardioncology and Second Opinion Division, European Institute of Oncology, Istituto di Ricovero e Cura a Carattere Scientifico (IRCCS), Via Ripamonti 435, 20141 Milan, Italy; carlo.cipolla@ieo.it; 15 Cardioncology Unit, European Institute of Oncology, Istituto di Ricovero e Cura a Carattere Scientifico (IRCCS), Via Ripamonti 435, 20141 Milan, Italy; daniela.cardinale@ieo.it; 16 Biomedical Research Institute, Foundation for Research and Technology–Hellas (FORTH), 45115 Ioannina, Greece; dimitris.fotiadis30@gmail.com; 17 Unit of Medical Technology and Intelligent Information Systems, Department of Materials Science and Engineering, University of Ioannina, 45110 Ioannina, Greece

**Keywords:** elderly, breast cancer, cardiotoxicity, frailty, heart failure

## Abstract

Cardiotoxicity is a leading cause of mortality in the growing populations of elderly breast cancer (BC) patients. Breast cancer treatment in the elderly is highly challenging due to its heterogeneous nature and the lack of specific evidence, as this population is usually underrepresented in randomized clinical trials. Decision making requires a comprehensive approach, considering the type and stage of BC, the patient’s overall health status, life expectancy, geriatric and frailty assessment, the risk of cancer recurrence, comorbidities, cardiotoxicity risk, and the patient’s preferences. The cardiotoxic effects of BC treatments cover the whole spectrum of cardiovascular diseases: heart failure, hypertension, arrhythmias, and myocardial ischemia. Cardiotoxicity risk in these patients is defined by several factors: anticancer therapies, polypharmacy, established cardiovascular disease, comorbidities, frailty, cellular senescence, hormonal changes, and genetic predisposition. Preventive oncological and cardio-oncological strategies, as well as patients’ education, are critical for improved outcomes. Prospective clinical trials in this population are urgently needed.

## Simple Summary

This review focuses on cardiotoxicity in elderly breast cancer patients. The factors that contribute to their high cardiotoxicity risk and the challenges in the management due to comorbidities and frailty are presented. It also includes specific recommendations from the oncology and cardio-oncology guidelines, preventive and educational strategies foroptimizing outcomes in this vulnerable population. Given the increasingnumber of elderly breast cancer patients and survivors and the limited available data, there is a critical need for prospective trials like the ongoing CARTIER and CARDIOCARE, that will facilitate the managementof this special population.

## 1. Introduction

Breast cancer (BC) has become the most prevalent form of cancer worldwide, surpassing lung cancer in 2020. It accounts for approximately 12.5% of all new annual cancer cases globally. Recent data reveal that global BC incidence has significantly increased since 1990, especially in older women [1], and BC is the second leading cause of death in women >60 years [2]. However, the 5-year relative survival rates by age is 92% in ages 65–74 and 86% in older adults [3]. One of the major concerns for the growing population of BC survivors, particularly older adults, is cardiotoxicity [4,5]. Breast cancer treatments, including chemotherapy, hormonal therapies, and radiotherapy, can have detrimental effects on the cardiovascular (CV) system, increasing the likelihood of heart failure (HF), coronary artery disease, and arrhythmias. Cardiovascular mortality mainly due to cardiotoxicity is the leading cause of death in older women with BC [6,7]. Despite growing awareness of cardiotoxicity risks, significant knowledge gaps remain, particularly due to the underrepresentation of elderly BC patients in clinical trials and the lack of age-specific data on cardioprotective strategies.

## 2. Treatment Options in the Elderly Breast Cancer Patients

Breast cancer treatment in the elderly is highly challenging due to its heterogeneous nature and the lack of specific evidence for older adults, as this population is usually underrepresented in randomized clinical trials. Breast cancer management in the elderly has largely been based on extrapolation of data from randomized controlled trials in younger patients. Effective decision making requires a comprehensive and holistic approach, considering multiple factors such as the type and stage of BC, the patient’s overall health status, life expectancy, competing risks of mortality, geriatric assessment, and frailty, the risk of cancer recurrence, patient’s preferences and the presence of comorbidities [8,9]. Comorbidities including hypertension, diabetes, HF, renal failure, and cognitive impairment, among others, may affect overall mortality, but also increase the risk of treatment-related toxicity and/or lead to non-adherence to therapy [10].

Additionally, physicians’ perception about biological and chronological age and poor tolerance or response to treatment or treatment-associated toxicity may result in biased decisions, sub-optimal implementation of guidelines and undertreatment [11,12,13,14,15]. Performance status alone may not be adequate to differentiate the heterogeneous older population with BC, and incorporation of geriatric assessment tools, including frailty assessment, to support appropriate therapy is recommended [9,16]. In addition, regular communication between healthcare providers, patients, and their families or caregivers is essential to ensure that the selected treatment plan aligns with the individual’s goals and preferences.

Treatment options for BC in older patients according to the “Updated recommendations regarding the management of older patients with breast cancer”, by the European Society of Breast Cancer Specialists (EUSOMA) and the International Society of Geriatric Oncology (SIOG), are presented in Table 1 [9].

In most early BC cases, surgery remains the primary treatment option in the elderly population [9]. Lumpectomy or mastectomy with sentinel node biopsy/axillary node clearance may be recommended, mostly depending on the stage of the disease [17]. For elderly patients who may have additional health concerns, a less aggressive surgical approach might be considered to minimize the impact on their overall well-being. Adjuvant therapies such as radiation therapy and systemic treatment may also be part of the management plan. Radiation therapy is employed to minimize the risk of local recurrence, while systemic therapy may be recommended to target any remaining microscopic disease. Endocrine therapy is a crucial aspect of BC treatment for hormone receptor-positive cancers both in the early and in the metastatic setting [18]. Due to presumed better tolerance, endocrine treatment may be considered as a primary treatment for hormone receptor-positive cases in the elderly [9], albeit adherence to oral treatment is considered a crucial problem in this age group due to polypharmacy, depression, refusal, or relevant comorbidities such as dementia.

Overall, the treatment of BC in the elderly necessitates a multidisciplinary approach focused not only on oncological outcomes but also on the patient’s overall health and personal preferences. Effective anticancer therapies may lead to the prolongation of survival up to several years, and during this time patients may suffer from both cancer-related complications and treatment-related toxicities, of which cardiotoxicity is the most crucial, affecting not only quality of life (QoL) but also morbidity and mortality.

## 3. Cardiotoxicity in Elderly Breast Cancer Patients

The cardiotoxic effects of BC treatments cover the whole spectrum of CV diseases (Table 2). They include myocardial dysfunction and HF, arrhythmias, myocardial ischemia, hypertension and metabolic impairments (e.g., increase in blood glucose, lipid levels, and body weight, leading to central obesity and physical deconditioning) [19,20] mainly induced by hormonal therapies.

### 3.1. Cancer Therapy Related Cardiac Dysfunction (CTRCD)

Cancer therapy related cardiac dysfunction (CTRCD), i.e., asymptomatic left ventricular dysfunction and HF, can be induced by nearly all antineoplastic agents used in BC treatment as well as by radiotherapy. The American Society of Clinical Oncology guidelines indicate a 1.6 to 6.8-fold increased risk of cardiac dysfunction in elderly patients (defined as 60 years of age or older) when compared to younger patients with cancer [21]. In a large study of early-stage BC patients aged 66–80 treated with anthracyclines, researchers found that every 10-year increase in age was associated with a 79% increase in the risk of congestive HF (hazard ratio 1.79, 95% CI 1.66–1.93) [22]. On the other hand, a study in elderly BC patients treated with trastuzumab demonstrated that the CTRCD risk was larger among patients 66–75 years old than those ≥76 (HR = 2.52 vs. 1.44) [23]. Severe cardiotoxicity correlates with a 10-fold increase in total mortality, according to the CARDIOTOX registry [24]. These findings underscore the need for age-specific cardiotoxicity risk assessment and suggest that older patients may require enhanced CV surveillance and more conservative oncologic strategies.

Anthracyclines are widely recognized as the most cardiotoxic class of antineoplastic agents, with doxorubicin and daunorubicin in particular being associated with a significant risk of cardiotoxicity and HF [25]. Epirubicin is structurally related to doxorubicin, but has shown lower cardiotoxicity risk [26], while pegylated liposomal doxorubicin demonstrates superior cardiac safety [27], particularly in older or frail patients with favorable long-term outcomes [28]. Anthracycline-induced cardiotoxicity is primarily a dose-dependent phenomenon, with the risk of cardiac dysfunction significantly increasing with higher cumulative doses. The risk of cardiotoxicity ranges from 3% to 4.7% at a cumulative doxorubicin dose of up to 400 mg/m^2^, increases to 7–26% at 550 mg/m^2^, and rises further to 18–48% at 700 mg/m^2^ [29,30]. Different anthracyclines exhibit varying degrees of cardiotoxicity, with the associated risk depending on both the specific agent and the administered dose. To facilitate risk assessment, equivalent cumulative doses can be estimated using established conversion factors that account for the relative cardiotoxic potential of each anthracycline [31]. Although doxorubicin use is associated with a three-fold increase in the rate of CTRCD in the first year after treatment, this risk is still 50% higher than the risk of patients who did not receive chemotherapy in the 5th year after diagnosis [32]. Age appears to be a significant factor in anthracycline-induced cardiotoxicity at equivalent cumulative doses. While 14.9% of individuals aged 40 to 59 experience cardiotoxic effects at a doxorubicin dose of 600 mg/m^2^, the incidence rises to 22.4% among patients over 60 years old receiving the same dosage [33]. Importantly, elderly patients have increased CTRCD risk even at lower cumulative doses of anthracyclines [30]. Furthermore, according to Pinder et al., BC patients between the ages of 66 and 70 who were treated with anthracyclines exhibited a 26% increased likelihood of developing congestive HF compared to those who received non-anthracycline therapies [22]. Anthracyclines seem to enhance the cellular senescence and telomere dysfunction which already exist in people of advanced age. Telomere dysfunction impairs mitochondrial biogenesis, further facilitating cellular senescence [34]. Other factors that contribute to the vulnerability of older hearts in cardiotoxicity is the age-related loss of cardiomyocytes, the decrease of myocardial volume and the altered pharmacokinetics of anthracyclines in the elderly with increased doxorubicin concentrations in the heart [35,36]. Increased risk (2 to 4 times) of CTRCD is also reported in older BC patients receiving trastuzumab [23,37], with an incidence of around 16.4% [38]. Previous or concurrent anthracycline use may increase this risk [31,38,39,40,41], but this is not confirmed by all studies [37].

However, although most of the studies and the recent European Society of Cardiology (ESC) cardio-oncology guidelines [31] use the term CTRCD mainly to describe asymptomatic left ventricular dysfunction and HF with reduced ejection fraction, there are data that indicate that the incidence of HF with preserved ejection fraction is higher in BC survivors than the incidence of HF with reduced ejection fraction (6.68% vs. 3.96%) [42]. Furthermore, mortality risk in hospitalized patients is higher for those with HF with preserved ejection fraction [42], revealing the importance of this entity. Supporting data for the increased incidence of HF with preserved ejection fraction in older BC patients and survivors come from a study in patients receiving contemporary radiotherapy, where HF with preserved ejection fraction is the predominant form of HF [43]. Interestingly, there is an increasing number of studies revealing right ventricular dysfunction in BC women [44,45,46,47,48,49,50], and the ESC and ESC Cardio-oncology Council strongly recommend assessing the right ventricle meticulously in cancer patients before and during treatment [31,51]. However, the majority of the data so far concern middle aged women [44,45,46,47,48,49,50]. The management of asymptomatic CTRCD or HF with reduced or with preserved ejection fraction induced by anticancer treatments follows the ESC 2022 Cardio-oncology Guidelines and the guidelines for HF in the general population, respectively [31,52], tailoring therapy according to the patient’s general status, preferences, and prognosis. Furthermore, in patients who develop early CTRCD during therapy, treatment adjustments should be carefully individualized, weighing oncologic efficacy against CV risk. Decisions regarding dose modification, temporary interruption, or therapy discontinuation or change should be made within a multidisciplinary team, following ESC, EUSOMA and SIOG recommendations [9,31].

### 3.2. Arrhythmias

Arrhythmias are one of the most frequent cardiotoxic events in BC patients and survivors, with an incidence of 11% after 15 years of follow-up [53] and a hazard ratio of 2.14 the first year after diagnosis. In anthracycline-treated BC patients, the incidence of arrhythmias is between 30–40% [53,54]. The incidence of these is low (3%) the first hour after the infusion but increases in the first 24 h (24%) [55]. Sinus bradycardia has been associated with cyclophosphamide, 5-fluorouracil, paclitaxel, and taxanes, while sinus tachycardia is associated with cyclophosphamide, 5-fluorouracil, paclitaxel, and epirubicin. Atrial fibrillation (AF) has been linked to the use of cyclophosphamide, doxorubicin, and taxanes [56]. Supraventricular tachycardias can occur with cyclophosphamide, doxorubicin, and taxanes, while premature ventricular contractions are associated with doxorubicin, taxanes, and 5- fluoruracil. Ventricular tachycardia or ventricular fibrillation may be observed with cyclophosphamide, 5-fluoruracil, doxorubicin, trastuzumab, taxanes, and tamoxifen. Atrioventricular block has been detected as a potential side effect of cyclophosphamide, doxorubicin, epirubicin, 5-fluoruracil, and taxanes. Prolongation of the QTc interval has been reported with doxorubicin, cyclophosphamide, 5-fluoruracil, paclitaxel, ribociclib, and tamoxifen; torsades de pointes arrhythmias have been linked to anthracyclines, 5-fluorouracil and tamoxifen [57,58,59,60,61]. Management of these arrhythmias should follow guideline-directed therapy as applied in the general population while tailoring decisions to the cancer type and stage, prognosis, drug–drug interactions, and individual patient preferences.

### 3.3. Myocardial Ischemia

Myocardial ischemia may be the result of age-related comorbidities in association with taxanes, pyrimidine analogues, alkylating agents, vinca alkaloids, endocrine therapies (aromatase inhibitors) and radiotherapy, especially for left-sided BC [19,62,63]. On the contrary, tamoxifen use has a significant protective effect on elderly BC women [64]. The risk of myocardial ischemia is increased only in the first year after diagnosis, while the long-term risk is not [53]. This can be attributed to the emotional stress that often accompanies the diagnosis of cancer and the increased peri-operative risk. Treatment of acute coronary syndromes in elderly BC patients can be challenging due to increased thrombotic and bleeding risks, comorbidities, frailty, and the possible need for other surgeries or interventions. However, the guidelines indicate that the recommendations for the general population can be applied, considering patient’s preferences, prognosis and performance status [31].

### 3.4. Hypertension

Hypertension can be induced or exacerbated by taxanes, alkylating agents, Vascular Endothelial Growth Factor inhibitors, Poly(ADP-ribose) polymerase inhibitors, endocrine therapies (aromatase inhibitors, selective estrogen receptor modulators), estrogen receptor downregulators, sequential or combination therapy (aromatase inhibitors plus CDK inhibitor) and left-sided radiotherapy [19,65,66]. Endothelial dysfunction, oxidative stress, imbalance between vasodilation and vasoconstriction, autonomic system dysfunction, decreased renal NO bioavailability, and decreased sodium excretion are some of the proposed underlying pathophysiological mechanisms [67,68]. In BC patients with pre-existing hypertension, the optimization of anti-hypertensive treatment before any cancer is advised. In cases of newly diagnosed hypertension or increased blood pressure values in previously well-controlled individuals, control of stress and pain, assessment of renal function, and counseling for a healthier diet with salt restriction and exercise should complement the administration of antihypertensives and precede the interruption of cancer treatment, if needed. Angiotensin receptor blockers or angiotensin-converting enzyme inhibitors are the first-line medications with or without a dihydropyridine calcium channel blocker, according to the baseline blood pressure values [31,65], in line with the recommendations for the general population. An individual patient’s risk factors will indicate the selection of the specific anti-hypertensive medication. In cases of resistant hypertension, b-blockers, spironolactone, and oral or transdermal nitrates or hydralazine can be considered. Interruption of cancer treatment is obligatory if systolic blood pressure is ≥180 mmHg or diastolic blood pressure ≥110 mmHg [31].

## 4. Cardiotoxicity Risk Factors in the Elderly

**Figure 1:**
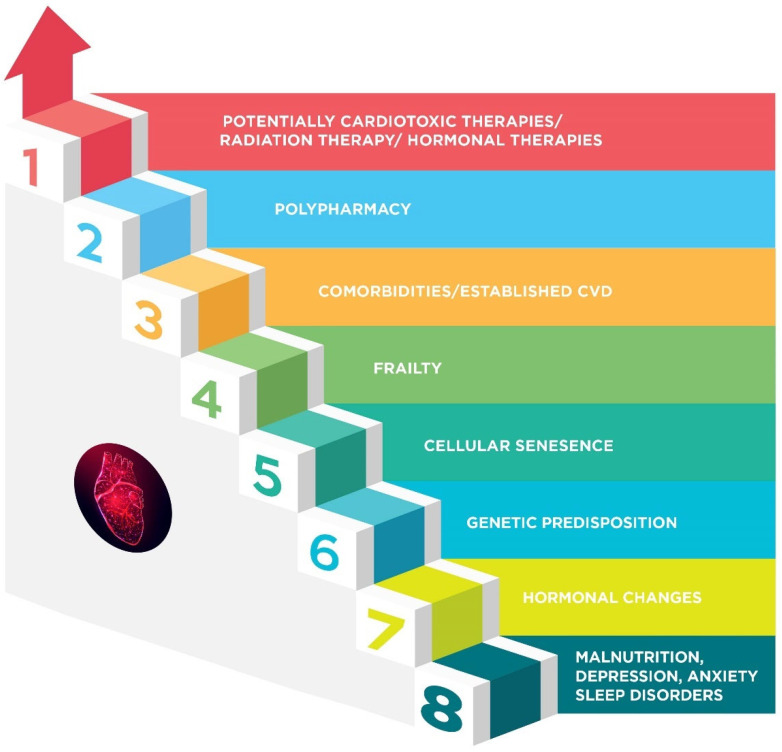
Overview of key risk factors contributing to cardiotoxicity in elderly breast cancer patients, including comorbidities, pre-existing cardiovascular conditions, potentially cardiotoxic therapies, frailty, polypharmacy, genetics, sleep disorders, psychological problems, and age-related physiological changes. CVD: cardiovascular disease.

Cardiotoxicity in the elderly is characterized by a complex terrain (Figure 1) that is shaped by several factors. Age itself makes people more susceptible to cardiotoxic chemotherapy [37]. This is partly ascribed to modifications in cardiac structure and function brought on by aging [69]. The aging heart experiences anatomical and functional changes affecting the resistance to strain brought on by specific treatments, and deteriorating cellular repair mechanisms and impaired arterial elastic properties [70] are additional contributing factors.

### 4.1. Frailty

Frailty, a condition marked by increased vulnerability to stressors due to diminished physiological reserve and resilience across multiple physiological systems [71], is a quite common condition in elderly patients. The prevalence and incidence of frailty in the elderly vary depending on the assessment tools used and the study population. In a recent meta-analysis including 62 countries and territories, frailty prevalence was 12% using the physical frailty phenotype definition and 24% using the deficit accumulation model [72]. Frailty is common in patients with cancer, with more than half of the older oncological population being pre-frail or frail [73]. These patients experience an increased risk of chemotherapy intolerance, postoperative complications, and all-cause mortality [73]. Moreover, recent data from 11,054 BC patients reveal that the incidence of cardiotoxicity is higher in pre-frail (12.5%) and frail (15.9%) patients compared to patients with no deficit (9.1%) (*p* < 0.001) [74]. Having recognized the prognostic importance of frailty, the updated recommendations of EUSOMA and the SIOG regarding the management of older patients with BC in 2021 recommend screening for frailty for patients aged ≥70 years to identify those at increased susceptibility to stressors and adverse outcomes [9].

### 4.2. Comorbidities and Established Cardiovascular Disease

The constellation of comorbidities and the increased incidence of established CV disease in older BC patients is an undeniable fact. Hypertension, diabetes, chronic renal disease, anemia, ischemic heart disease, valvular disease, HF, dementia, and cognitive impairment may interact with BC therapies that are potentially cardiotoxic, increasing the likelihood of unfavorable cardiac events [75]. Moreover, the polypharmacy that accompanies the aforementioned conditions has the potential not only to decrease adherence to treatment but also to increase cardiotoxic effects due to drug–drug interactions.

### 4.3. Hormonal Changes

Hormonal variables can also increase cardiotoxicity risk. Age-related hormonal changes, such as those that occur during menopause, lead in CV aging [76]. Estrogen, progesterone, and androgen decline enhance CV risk by means of endothelial dysfunction, arterial stiffening, cardiac remodeling, and unfavorable metabolic changes including increases in LDL cholesterol and decreases in HDL cholesterol, insulin resistance, and impaired glucose metabolism [76]. These adverse changes make elderly cancer patients and survivors more vulnerable to cardiotoxic effects from antineoplastic therapies, including endocrine therapies that further increase CV morbidity and mortality [19].

### 4.4. Genetics

The role of genes in making a patient vulnerable to CTRCD, especially from anthracyclines, is recognized more and more. Genetic susceptibility to CTRCD is influenced by polymorphisms in genes related to drug metabolism, oxidative stress, DNA repair, and cardiomyocyte function, but also by epigenetic changes [77]. Variants in anthracycline metabolism genes (e.g., ABCB1, ABCC1, NQO1) can affect drug accumulation in cardiomyocytes, influencing toxicity [78,79]. Additionally, polymorphisms in oxidative stress genes (SOD2, GSTP1) and genes regulating myocardial energy pathways (RYR2, TNNT2) have been linked to increased cardiotoxic risk [78,79,80,81]. In addition, associations have been found in the *p53* gene involved in regulating apoptosis and autophagy in response to oxidative stress and DNA damage (OR 2.972) and the *NOS3* gene involved in regulating blood vessel functioning (OR 3.059) [82]. Several *HER2* gene polymorphisms have been linked to trastuzumab-induced cardiotoxicity, with the strongest associations seen in the single nucleotide polymorphisms (SNP) *HER2* 655 isoleucine/valine [83,84] and the *HER2* 1170 proline/alanine [85]. Many individual SNPs have also been associated with cardiotoxicity through genome wide association studies (GWAS) [86]. While several studies associate different polymorphisms with increased risks of cardiotoxicity, replication across large adult cohorts remains limited.

### 4.5. Malnutrition, Psychological, and Sleep Disorders

Several additional factors common in elderly cancer patients may contribute to the development of cardiotoxicity. Nutritional deficiencies or imbalances, often prevalent in older populations, can exacerbate vulnerability to cardiotoxic effects [87]. Malnutrition weakens cardiac and systemic resilience, impairs wound healing, and increases susceptibility to infections and treatment-related complications [88]. Psychological and social factors may also play a role in cardiotoxicity of anticancer treatments. Depression, anxiety, and social isolation are prevalent among elderly cancer patients [89] and have been shown to negatively impact CV health by promoting unhealthy behaviors, reducing adherence to treatment, and increasing stress-mediated physiological responses [90,91]. Anxiety diagnosed prior to BC increases the risk of CV disease in BC survivors [92]. Sleep disorders, including insomnia and obstructive sleep apnea, are frequently underdiagnosed in the elderly population [93,94] and increase the risk of CV disease in cancer patients [95,96]. Obstructive sleep apnea in cancer patients increases their risk of developing arterial hypertension [97], HF [98,99], atrial fibrillation, atrial flutter, myocardial infarction, and ischemic stroke [99].

## 5. Preventive Strategies

The prevention of cardiotoxicity in elderly patients requires close collaboration among the involved medical disciplines, crucially the oncologists and the cardiologists but also the geriatric specialists, if available (graphical abstract).

### 5.1. Cardio-Oncological Strategies

Cancer patients managed in dedicated cardio-oncology clinics by well-trained cardiologists in cardio-oncology often experience better prognoses due to a comprehensive and multidisciplinary approach [100]. These clinics provide specialized care focused on the early detection, prevention, and management of cardiotoxicity, ensuring that CV health is prioritized without compromising cancer treatment [101,102]. The main aim of the cardio-oncology contribution is to minimize treatment interruptions, improve patient outcomes, and enhance overall QoL.

**Figure 2:**
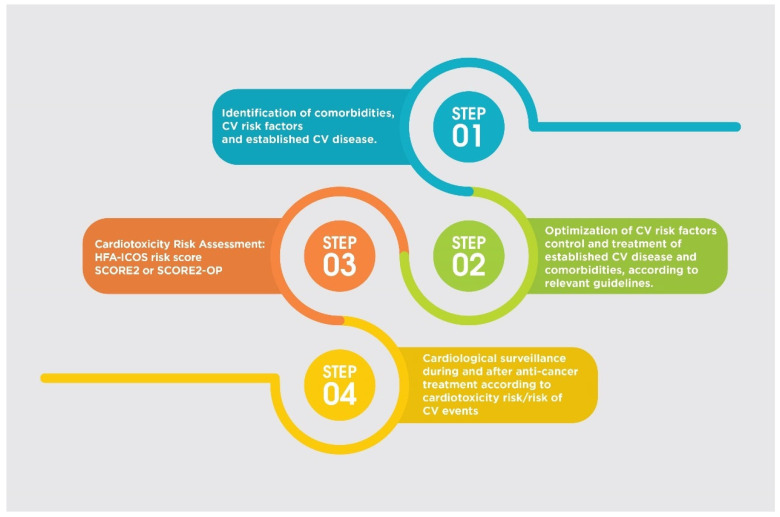
Multistep prevention strategy for cardiotoxicity in elderly breast cancer patients. This figure illustrates a four-step approach to minimize cardiovascular (CV) risk during cancer treatment in elderly patients. Step 1 involves the identification of comorbidities, CV risk factors, and established CV disease. Step 2 focuses on optimizing the management of CV risk factors and treating existing CV conditions according to current guidelines. Step 3 emphasizes cardiotoxicity risk assessment using validated tools, such as the HFA-ICOS risk score, SCORE2, or SCORE2-OP. Step 4 outlines the need for cardiological surveillance during and after cancer therapy, tailored to the patient’s cardiotoxicity and CV risk profile.

Baseline CV assessment is of critical importance to prevent the development of cardiotoxicity and to ensure the best possible outcome both for cancer treatment and for the CV system. Identification of comorbidities, CV risk factors, and established CV disease is the first important step. The second one is the optimal control of these conditions by applying guideline-recommended treatments for primary and secondary prevention of CV disease. The third step is a cardiotoxicity risk assessment by calculating an HFA-ICOS risk score for certain drug categories (classical chemotherapeutics and HER2 (human epidermal growth factor receptor 2) targeted therapies) and of the 10-year fatal and non-fatal CV disease risk by SCORE2 (in patients <70 years old without clinical manifestations of atherosclerotic disease) and SCORE2-OP (if ≥70 years) in patients scheduled to receive hormonal therapies [31]. Age ≥80 is a high severity risk factor, while age 65–79 is a moderate severity risk factor for patients that will receive anthracyclines and/or HER2 targeted therapies. For Vascular Endothelial Growth Factor inhibitors, age ≥75 is a high severity risk factor and age 65–74 is a moderate severity risk factor. So, taking together frailty and the common comorbidities, the majority of older patients have a moderate to high baseline cardiotoxicity risk and require close cardiological follow-up during and after BC treatment (Figure 2). In patients at high and very high cardiotoxicity risk, cardioprotective therapies may be given before any cancer treatment, as neurohormonal therapies including renin–angiotensin–aldosterone system blockers, beta-blockers, and mineralocorticoid receptor antagonists have shown favorable effects in preserving left ventricular ejection fraction during anthracycline chemotherapy and HER2-targeted therapies [103,104,105,106,107,108]. However, most of these trials have included mixed-age populations and were not specifically powered for elderly subgroups. Notably, a study by Wittayanukorn et al. directly addressed this gap by focusing on older adults with BC. In this large retrospective cohort of women aged ≥66 years receiving trastuzumab and/or anthracyclines, the initiation of angiotensin-converting enzyme inhibitors or beta-blockers was associated with a 23% reduction in cardiotoxicity and a 21% reduction in mortality [109].

### 5.2. Oncological Strategies

All management decisions for elderly patients with BC should follow a thorough geriatric assessment and consider physiological age, life expectancy, potential risks versus absolute benefits, CTRCD risk, frailty, treatment tolerance, patient preferences, and potential barriers to treatment [110]. Treatment decisions for anticancer treatment should be based not only on the risk of recurrence or BC mortality but should also weigh the risk of dying of other causes, e.g., HF, as an equally important factor. Having recognized the treatment challenges in this special population EUSOMA and the SIOG have recently published specific recommendations Table 1 [9]. Key points are the following:

✓ Anthracyclines can be avoided in high- and very high-cardiotoxicity risk patients. Only carefully selected, fit, older patients with high-risk disease (large, node-positive, triple-negative) can be considered for a sequential combination of anthracyclines and taxanes [9].
✓ Limitation of the cumulative anthracycline dose as the risk of CTRCD is dose-dependent [111].
✓ Selection of epirubicin instead of doxorubicin, as it is less cardiotoxic than doxorubicin [112].
✓ Prolonged administration of doxorubicin (continuous infusion rather than bolus administration, as it has been associated with a lower rate of HF) [113,114].
✓ Administration of weekly divided doses of anthracyclines significantly decreases CV damage without affecting its anticancer efficacy [114,115].
✓ Liposomal doxorubicin that has been approved for metastatic BC can be used instead of unencapsulated anthracycline formulations with a significantly lower cardiotoxicity risk [116].
✓ Dexrazoxane is a cardioprotective agent that has a proven cardioprotective effect [106,117] and is formally approved in adult patients with advanced or metastatic BC who have already received a minimum cumulative anthracycline dose of 300 mg/m^2^ of doxorubicin or 600 mg/m^2^ of epirubicin or equivalent [118,119].
✓ Weekly paclitaxel (for 12 weeks) can be an option in patients unfit for polychemotherapy [9].
✓ Shorter courses of chemotherapy or HER2-targeted therapies can be applied in high-risk older patients [9].

### 5.3. Patient Education

Educating patients about the potential CV risks associated with therapies such as anthracyclines, HER2-targeted agents, or radiation allows them to recognize early warning signs of cardiotoxicity, such as shortness of breath, palpitations, chest pain, edema, or fatigue. Lifestyle changes, including adopting a heart-healthy diet rich in fruits, vegetables, whole grains, and lean proteins, play a vital role in reducing CV risk factors. Regular physical activity tailored to the patient’s capacity, such as walking or light aerobic exercises, can improve CV fitness and mitigate treatment-related fatigue [119,120,121]. Smoking cessation and moderation of alcohol consumption are also essential to lower the risk of cardiotoxicity [122]. Patients should also be encouraged to maintain a healthy weight and attend regular follow-ups with both their oncologist and cardiologist to monitor cardiac function, enabling timely intervention if cardiotoxicity arises. The ESC and ESC Cardio-oncology Council, having realized the critical role of patient education, published the “ESC Clinical Practice Guidelines on Cardio-oncology: What the patient needs to know” in 2022 [123]. Empowering patients through education and emphasizing lifestyle modifications provides a proactive approach to safeguarding CV health during cancer treatment.

## 6. Evolution of Cardiotoxicity Prevention in Elderly Breast Cancer Patients and Clinical Trials

Unfortunately, older BC patients are often underrepresented in clinical trials, though a few of them are dedicated to elderly patients. Even these however, are mostly retrospective and limited to the assessment of the risk or the incidence of cardiotoxicity in this population and to the predictors associated with cardiotoxicity (age, hypertension, diabetes, coronary artery disease, concomitant use of anthracyclines and trastuzumab, black race, etc.) [22,32,41,70,124,125,126,127]. CAPRICE is a prospective phase II trial that evaluated neoadjuvant pegylated liposomal doxorubicin in elderly patients or in those with other CV risk factors in whom conventional doxorubicin was contraindicated [28]. This treatment option proved to be safe concerning cardiotoxicity risk and effective in this fragile population.

The risk of developing HF or asymptomatic left ventricular dysfunction is higher in the older studies compared to the most recent ones, revealing the evolution of cardio-oncology and a higher awareness among clinicians, leading to improvements in surveillance, prevention, and treatment strategies.

Two ongoing prospective trials, CARTIER and CARDIOCARE, aim to assess preventive strategies for cardiotoxicity in elderly cancer patients. CARTIER is a randomized, multicenter, open-label clinical trial designed to compare two cardiotoxicity prevention strategies (primary vs. secondary) in elderly patients (>65 years) with specific onco-hematological cancers (colon, breast, lymphoma, chronic lymphocytic leukemia, chronic myeloid leukemia, or myeloma) [128]. The primary endpoint is to determine whether primary prevention, which includes intensive CV monitoring and multidisciplinary management by cardio-onco-hematology teams, is superior to standard care in reducing all-cause mortality. Secondary outcomes include the incidence of CV and oncologic mortality, hospitalizations due to cardiac or cancer complications, tumor progression, and a cost-effectiveness analysis. A total of 514 patients will be followed for 2 and 5 years, with scheduled CV assessments at baseline, 3, and 6 months, and annually thereafter. The trial’s outcomes are expected to inform future recommendations for the routine implementation of risk-adapted cardiotoxicity prevention and multidisciplinary management in elderly cancer patients.

On the other hand, CARDIOCARE is an observational prospective trial dedicated to elderly BC patients that aims to refine and validate risk stratification algorithms for the development of cardiotoxicity not only of the left but also of the right ventricle. Cardiotoxicity related to cancer therapy in this trial is identified according to the most recent definition included in the ESC 2022 Cardio-oncology Guidelines [31], which is based not only on changes of left ventricular ejection fraction and myocardial strain but also of traditional biomarkers (i.e., troponin and natriuretic peptides). Novel biomarkers, such as single nucleotide polymorphisms, microRNA panels, and gut microbiome bacterial profiles, are also assessed in this population in order to identify patients susceptible to the development of CTRCD [129]. Eligible participants are women aged ≥60 years undergoing neoadjuvant or adjuvant therapy with anthracyclines, taxanes, endocrine therapy ± CDK4/6 inhibitors, or anti-HER2 agents in both early and metastatic BC settings. In addition to standard care, all participants receive supportive digital health monitoring, including wearable devices and completion of the ePsycHeart mobile evaluation, which assesses domains such as psychocognitive function, mobility, vitality, and sleep. An innovative aspect of CARDIOCARE is the integration of a digital behavioral and psychological intervention platform (eHealtHeart). Patients in the intervention arm receive personalized strategies targeting emotional well-being, cognitive resilience, physical performance, nutrition, and caregiver support designed to mitigate or delay cardiotoxicity and improve QoL. The primary objective is to assess the incidence of subclinical and clinical cardiotoxicity, while secondary endpoints include MACEs, deterioration of intrinsic capacity, functional decline, and QoL outcomes. [130]. Its findings will likely contribute to the refinement of clinical risk assessment tools and the integration of supportive behavioral interventions into standard cardio-oncology care for older patients.

Future research in elderly patients should focus on higher representation of this population in clinical trials with appropriate stratification for functional status, integration of systematic geriatric assessment, including frailty and comorbidities to produce age-specific efficacy and safety data for systemic therapies. Another area of further potential research is that of comparative trials evaluating standard versus de-escalated regimens in the elderly; for example, shorter duration of specific drugs, reduced dosing, and/or omission of chemotherapy in favor of endocrine therapy alone. Of critical importance is to design clinical trials focusing on or incorporating assessment of QoL and independence, as these are often sacrificed for survival. The paucity of data on areas such as cardiotoxicity or cognitive decline limit oncologists’ ability to effectively recognize and ultimately manage such conditions. Furthermore, future clinical trials should produce evidence for the management of specific subtypes (HR positive, TNBC, Her2 positive disease) adopting the precision medicine paradigm (biomarker driven) to improve the outcomes of elderly patients, particularly those with aggressive subtypes. More trials like PRIME II, focusing on radiotherapy de-escalation or omission or hypofractionation in fit versus frail elderly patients, could provide important results [131]. Finally, as randomized controlled trials may often not reflect routine practice and patient diversity, more real-world trials should be performed in the elderly population to tackle this problem.

## 7. Conclusions

Elderly patients with BC comprise a rapidly growing population with special needs driven by the high morbidity and mortality and the elevated cardiotoxicity risk due to the complex interplay of comorbidities, frailty, and age-related physiological changes, pre-existing CV risk factors and disease, and polypharmacy. Treatment decisions in these patients should be guided by the increased mortality risk not only of cancer, but also of CV and other causes related to aging. Preventive strategies include cardiological and oncological measures combined with patient education and active participation in decision making throughout cancer treatment. Further prospective, elderly-focused research is urgently needed to address current gaps in evidence, especially regarding specific biomarkers for cardiotoxicity prediction, individualized treatment strategies, cardioprotection, and optimal surveillance protocols in this age group.

## Figures and Tables

**Table 1:** Treatment options for breast cancer in older patients according to the 2021 recommendations by the European Society of Breast Cancer Specialists (EUSOMA) and the International Society of Geriatric Oncology (SIOG).

Type of Treatment	2021 Recommendations by EUSOMA–SIOG
**Surgery**	▪ Surgery remains the choice of primary treatment in the majority of older patients with early breast cancer (level 1) ▪ For patients with a positive sentinel lymph node, completion axillary therapy (surgery or radiotherapy) is not always needed, and if needed, radiotherapy should be preferred to axillary clearance, especially in the cases of low axillary nodal burden and ER-positive disease requiring adjuvant endocrine therapy (level 4) ▪ Axillary surgery can be omitted in patients with cT1N0 luminal A-like tumors or short life expectancy (level 4) ▪ Surgery for DCIS should consider grade and life expectancy (level 4)
**Primary endocrine therapy**	▪ Primary endocrine therapy can be considered as an alternative in selected patients with a strongly ER-positive tumor and short life expectancy (no more than 5 years)
**Radiotherapy**	▪ WBRT remains the standard of care for most older patients following BCS, and omission of radiotherapy in low-risk patients can be safe and reasonable (level 1) ▪ In patients >60 years, the use of a boost is advised only for those at higher risk of recurrence (level 1) ▪ PBI is recommended to women ≥50 years and grade 1–2, pN0, hormone receptor-positive, HER2-negative, tumors ≤30 mm with radial margins ≥1 mm (level 4); the role of postmastectomy radiotherapy in patients with one to three positive nodes remains controversial ▪ Hypofractionated schedules (40 Gy in 15 fractions over 3 weeks, 42.5 Gy in 16 fractions over 3.5 weeks or 26 Gy in five fractions over 1 week) are recommended for older patients (level 4)
**Adjuvant chemotherapy in HER2-negative disease**	▪ Older adults with hormone receptor-negative disease can derive the most benefit from adjuvant chemotherapy irrespective of nodal status (level 3) ▪ Standard regimens include four cycles of docetaxel–cyclophosphamide or four cycles of doxorubicin and cyclophosphamide (level 2) ▪ Weekly paclitaxel (for 12 weeks) can be an option in patients unfit for polychemotherapy (level 4) ▪ Only carefully selected, fit, older patients with high-risk disease (large, node-positive, triple-negative) can be considered for a sequential combination of anthracyclines and taxanes (level 4)
**Adjuvant anti-HER2 therapy**	▪ Adjuvant chemotherapy along with one year of trastuzumab is recommended as a standard approach in older patients with no cardiac dysfunction and early-stage, HER2-positive breast cancer ≥0.5 cm (level 2) ▪ Preferred chemotherapy options: taxanes without anthracyclines, for example in the form of four cycles of docetaxel–cyclophosphamide or 12 consecutive weeks of weekly paclitaxel, avoiding the cardiac toxicity of anthracyclines and duration of chemotherapy beyond the 3-month threshold at risk of grade 3–5 adverse events (level 4) ▪ A sequential regimen of anthracyclines and taxanes with trastuzumab is appropriate only in a very selected group of fit, healthy older patients (level 4) ▪ Pertuzumab can be added only in high-risk and fit patients, but diarrhea can be a debilitating side effect in older individuals (level 4) ▪ Although evidence is scarce, the use of single-drug trastuzumab without chemotherapy, but with endocrine therapy if hormone sensitive, can be appropriate in susceptible and frail patients (level 4) ▪ Shorter courses of anti-HER2 therapy can be considered for older patients with small, node-negative tumors or in the context of cardiac problems (level 2)
**Adjuvant endocrine therapy**	▪ Aromatase inhibitors are slightly more beneficial than tamoxifen with regards to the risk of recurrence and breast cancer mortality and should be considered the standard of care in older women (level 4)
**Chemotherapy (metastatic breast cancer)**	▪ All available chemotherapeutics can be used in principle like in younger people, some evidence suggests the use of single drug nab-paclitaxel and eribulin in older patients (level 2)
**HER2-positive disease (metastatic breast cancer)**	▪ Anti-HER2 therapy should be given unless contraindicated by impaired left ventricular ejection fraction, with treatment adjusted according to patient fitness (level 1) ▪ A taxane, preferably paclitaxel, in combination with trastuzumab and pertuzumab is recommended as first-line therapy only in fit patients, as it can cause unacceptable toxicity in patients who are unfit (level 4); ▪ Endocrine therapy can be suitable in lieu of chemotherapy in patients with hormone receptor-positive disease (level II) ▪ In patients who are unfit, taxane-free chemotherapy backbones include metronomic cyclophosphamide, vinorelbine or capecitabine (level 2) ▪ Trastuzumab can be used in second line or later lines of therapy in fit patients, with careful monitoring in patients who are frail (level 4)
**Targeted therapies**	▪ CDK4/6 inhibitors in combination with endocrine therapy represent a suitable treatment in older patients, with frequent adjustments needed (level 3) ▪ Endocrine therapy alone is still a reasonable first-line option in selected cases (level 3) ▪ Use of everolimus should be approached with caution and on a case-by-case basis due to its worse safety profile in older patients (level 2)
**Adjuvant bone modifying agents**	▪ Adjuvant bisphosphonates (either zoledronic acid 4 mg every 6 months or clodronate 1600 mg daily) should be offered to patients with moderate-risk to high-risk disease, regardless of age (level 4)

BCS: Breast Conserving Surgery, CDK4/6: Cycline-dependent Kinase 4/6, DCIS: Ductal Cancer In-Situ, ER: Estrogen Receptor, Gy: Gray, HER2: human epidermal growth factor receptor 2, WBRT: Whole Breast Radiotherapy.

**Table 2:** Cardiotoxicities of Breast Cancer treatments.

Types of Breast Cancer Treatment	Cardiotoxicities
**Anthracyclines** *e.g. doxorubicin*	HF or asymptomatic LVD or RVD Arrhythmias, Takotsubo syndrome
**Taxanes** *e.g. docetaxel, nab-paclitaxel*	Arrhythmias, conduction disorders myocardial ischemia, hypertension
**Vinca alkaloids** *e.g. vinorelbine*	Myocardial ischemia, AF
**Antimetabolites** Pyrimidine analogues *e.g. 5-Fluoruracil, capecitabine*	Myocardial ischemia, HF, or asymptomatic LVD
**Alkylating agents** *e.g. cyclophosphamide, carboplatin, oxaliplatin*	Myocardial ischemia, HF, or asymptomatic LVD, hypertension, pericarditis, myocarditis, arrhythmias
**HER2 targeted therapies**	HF or asymptomatic LVD or RVD
**Monoclonal antibodies** *e.g. trastuzumab, pertuzumab*	
**Dual blockade** *e.g. trastuzumab + pertuzumab*	Hypertension
**Antibody Drug Conjugates (ADCs)** *Trastuzumab emtansine (TD-M1)* *Sacituzumab govitecan* *Trastuzumab deruxtecan (T-DXd)*	LVD, QTC prolongation
**HER2 TKIs** Neratinib, tucatinib, lapatinib	LVD, Prinzmetal’s angina
**Poly (ADP-ribose) polymerase inhibitors** *e.g. niraparib*	Hypertension
**mTOR inhibitors** *e.g. everolimus, sirolimus*	Hypertension
**Anti-VEGF** *e.g. bevacizumab*	Hypertension
**Immune checkpoint inhibitors** *e.g. pembrolizumab*	New onset hypertension, stable angina, acute HF, Myocarditis <1%, arrhythmias
**Microtubule dynamics inhibitor** *e.g. Eribulin*	QTc prolongation
**PARP inhibitors** *Olaparib, Talazoparib*	MACEs, hypertension, thromboembolic events
**CDK4/6 inhibitors** *abemaciclib, palbociclib, ribociclib. dalbiciclib*	Thromboembolic events, QTc prolongation, LVD, HF, AF
**Endocrine therapy** *Aromatase inhibitors (e.g. anastrozole, letrozole, exemestane)*	Myocardial ischemia, HF, hypertension, dyslipidemia
*Estrogen Receptor Downregulators (e.g. fulvestrant)*	Hypertension
*Selective Estrogen Receptors Modulators (e.g. tamoxifen)*	VTE, ↑ triglycerides, diabetes risk, body fat
**Radiotherapy**	Myocardial ischemia, valvular disease pericarditis, HF, or asymptomatic LVD conduction abnormalities, hypertension
**Adjuvant bone modifying agents** **(bisphosphonates:** *zoledronic acid, clodronate)*	AF

AF: atrial fibrillation, HF: heart failure, MACEs: major cardiovascular events, LVD: left ventricular dysfunction, RVD: right ventricular dysfunction, VEGF: Vascular Endothelial Growth Factor, VTE: venous thromboembolism.

## Abbreviations

The following abbreviations are used in this manuscript:

BC	breast cancer
CV	cardiovascular
HER	human epidermal growth factor receptor
HF	heart failure
EUSOMA	European Society of Breast Cancer Specialists
SIOG	International Society of Geriatric Oncology
QoL	quality of life
CTRCD	Cancer therapy related cardiac dysfunction
ESC	European Society of Cardiology

## Appendix A

CARDIOCARE Consortium investigators: Gabriella Pravettoni: European Institute of Oncology, IRCCS, Milano, Italy and Department of Oncology and Hemato-oncology, University of Milan, Italy. Alexia Alexandraki: A.G. Leventis Clinical Trials Unit, Bank of Cyprus Oncology Centre, 32 Acropoleos Avenue, Nicosia 2006, Cyprus. Elisavet Fotiou: A.G. Leventis Clinical Trials Unit, Bank of Cyprus Oncology Centre, 32 Acropoleos Avenue, Nicosia 2006, Cyprus. Aristofania Simatou, 2nd Department of Oncology, General Anti-Cancer Oncological Hospital, Agios Savvas, 11522 Athens, Greece. Sofia Talagani, 1st Department of Oncology, General Anti-Cancer Oncological Hospital, Agios Savvas, 11522 Athens, Greece. Alexandros Mitsis: Unit of Medical Technology and Intelligent Information Systems, Dept. of Material Science and Engineering, University of Ioannina, GR45110 Ioannina, Greece. George C Manikis: Computational BioMedicine Laboratory (CBML), Institute of Computer Science, Foundation for Research and Technology Hellas (FORTH), 70013 Heraklion, Greece and Department of Oncology-Pathology Karolinska Institutet. Kostas Marias: Department of Electrical and Computer Engineering, Hellenic Mediterranean University, 71410 Heraklion, Greece and Computational BioMedicine Laboratory (CBML), Institute of Computer Science, Foundation for Research and Technology Hellas (FORTH), 70013 Heraklion, Greece. Katerina K. Naka: 2nd Cardiology Department, University of Ioannina Medical School, 45110 Ioannina, Greece. Lampros Lakkas: Physiology Department, Faculty of Medicine, School of Health Sciences, University of Ioannina, 45110 Ioannina, Greece. Grigorios Kalliatakis: Computational BioMedicine Laboratory (CBML) of Institute of Computer Science, Foundation for Research and Technology-Hellas (FORTH). Constanza Conti: Istituto di Management Sanitario S.r.l, Milano, Italy. Anca Bucur: Philips Research Europe, Eindhoven, Netherlands.
